# The perfused liver utilisation study (PLUS): challenges and innovation in the evaluation of an emerging technology

**DOI:** 10.3389/ti.2026.16653

**Published:** 2026-09-08

**Authors:** Simon Robert Knight, Helen L. Thomas, Rosemary Brown, Charlie Brown, Kerrie Brusby, Emily Arbon, Laura Silsby, Alice Newton, Jennifer Mehew, Aleema Iqbal, Katie Keen, Syeda Bahar, Christopher Watson, Brian Davidson, David Nasralla, Carlo Ceresa, Barbara Fiore, Ahmed E. Sherif, Colin Wilson, Balaji Mahendran, Joerg-Matthias Pollok, Rebeca S. Mateos, Wayel Jassem, Rohit Gaurav, Gabriel C. Oniscu, Craig Marshall, Angus Hann, Reena Ravikumar, Constantin Coussios, Peter J. Friend

**Affiliations:** 1 University of Oxford, Oxford, United Kingdom; 2 NHS Blood and Transplant, Bristol, United Kingdom; 3 University of Cambridge, Cambridge, United Kingdom; 4 University College London, London, United Kingdom; 5 Royal Free London NHS Foundation Trust, London, United Kingdom; 6 Leeds Teaching Hospitals NHS Trust, Leeds, United Kingdom; 7 NHS Lothian, Edinburgh, United Kingdom; 8 Newcastle Upon Tyne Hospitals NHS Foundation Trust, Newcastle uponTyne, United Kingdom; 9 University Hospitals Birmingham NHS Foundation Trust, Birmingham, United Kingdom; 10 King’s College London, London, United Kingdom; 11 Cambridge University Hospitals NHS Foundation Trust, Cambridge, United Kingdom; 12 Karolinska Institutet, Stockholm, Sweden; 13 Organox Limited, Oxford, United Kingdom

**Keywords:** clinical study, liver transplantation, normothermic machine perfusion, organ utilization, threshold crossing

## Abstract

Previous studies suggest that donor liver normothermic machine perfusion (NMP) reduces injury and enables functional assessment, resulting in an increase in utilisation. The PLUS study was designed to assess the impact of NMP availability on utilisation of livers at high risk of non-use. Using a threshold-crossing design, functional utilisation was assessed in a real-world retrospective registry-based control cohort. A predetermined efficacy threshold was set, and a prospective cohort was recruited in all UK liver transplant centres with NMP made available for all livers at high risk of non-utilisation. Availability of NMP resulted in a significant increase in functional utilisation (OR: 1.26, 95% CI: 1.04–1.53), but this did not exceed the pre-determined efficacy threshold. The threshold was exceeded in the subgroup of donation after circulatory death (DCD) livers, although this increase was reduced with adjustment for the use of normothermic regional perfusion. NMP was used in only 31% of retrieved livers, with variation between centres. The benefit of having NMP available was less than anticipated, likely due to low use in some centres associated with staff availability. Provision of NMP as a service, or centralisation of NMP use in assessment and recovery centres, may improve uptake.

## Graphical Abstract

**Figure:**
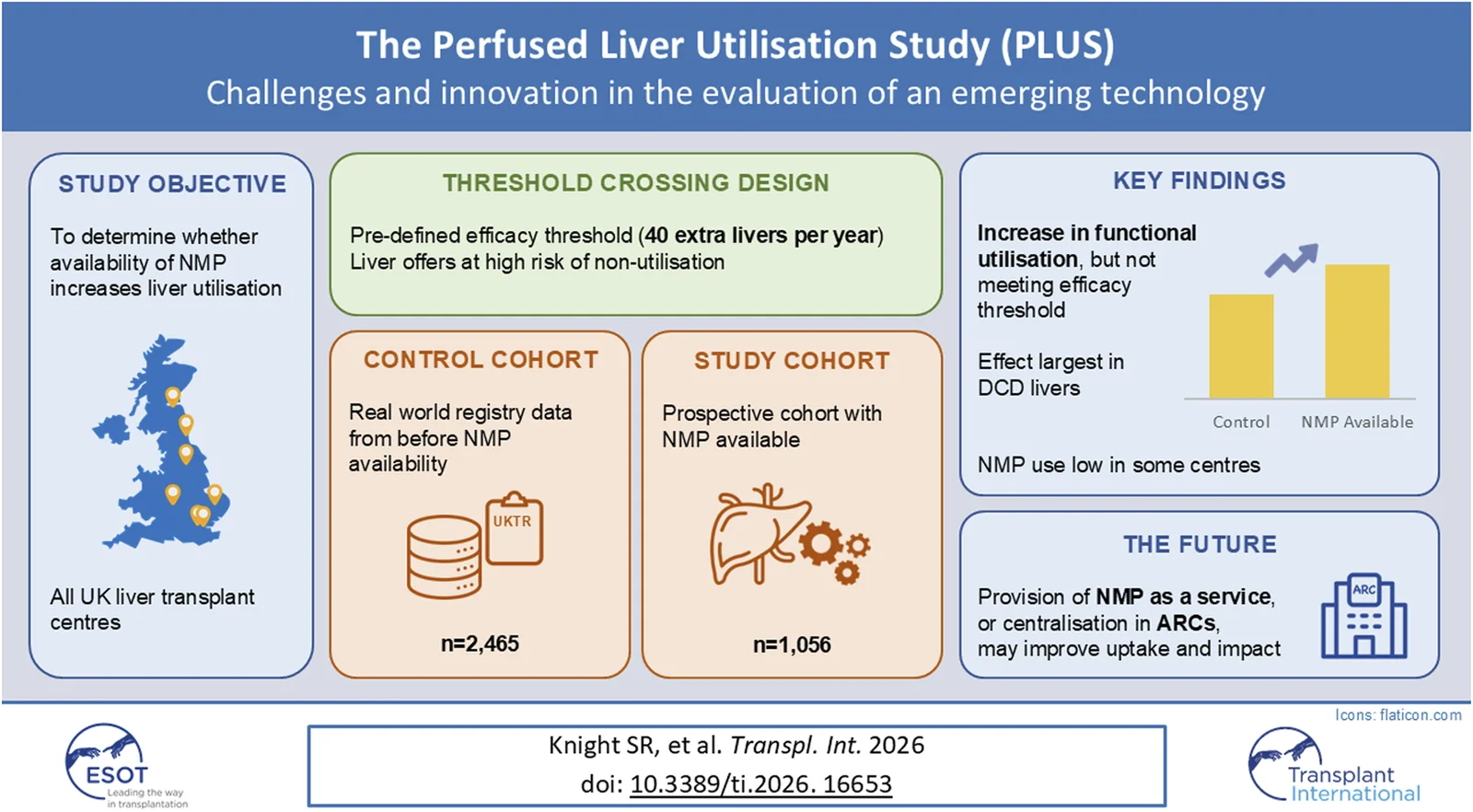

## Introduction

Liver transplant numbers in the UK have remained largely static over the past 10 years, whilst the number of patients waiting for liver transplantation has increased by 11% [1]. Around 70 patients die on the UK liver transplant waiting list each year. Only 57% of offered livers are transplanted, largely due to an increasing proportion of ‘high-risk’ organs and the lack of reliable preoperative viability assessment [2]. This is not just a UK phenomenon – this discrepancy between supply and demand is an international concern.

This organ shortage has led to increased interest in interventions with the potential to increase organ utilisation. Machine perfusion, either *in-situ* (normothermic regional perfusion; NRP) or *ex-situ* (normothermic or hypothermic machine perfusion) offers potential for reconditioning and functional assessment prior to transplant [3–5]. There is an increasing view that the ability to assess function *ex-situ* by means of normothermic machine perfusion (NMP) may improve confidence in transplanting more marginal organs, improving utilisation.

Randomised controlled trials comparing NMP to static cold storage (SCS) have shown reduced early allograft dysfunction with lower incidence of reperfusion injury, especially for higher risk livers such as those from donors after circulatory death (DCD) [6, 7]. The pivotal multicentre European randomised controlled trial also demonstrated an increase in utilisation of retrieved livers with NMP (non-utilisation 11.7% vs. 24.1%), despite eligibility for the trial requiring all livers to be suitable for either preservation method [6]. This suggests that clinicians were using the technology to assess the suitability of livers prior to transplantation, improving confidence and utilisation without compromising post-transplant outcomes. Registry-based studies from the US have demonstrated similar improvements in liver utilisation with NMP, particularly from DCD donors, although historically the utilisation of DCD livers in the US has been lower than in European countries [8, 9]. The benefit of NMP appears to be greatest for patients with low model for end-stage liver disease (MELD) scores [10]. Implementation of NMP may also have the potential to reduce waiting times, particularly for patients with hepatocellular carcinoma [11]. Small-scale studies, including most notably the VITTAL study from Birmingham [12], suggest that a meaningful proportion of donor livers that are currently declined on conventional criteria could in fact be transplanted successfully.

Despite this potential beneficial effect of NMP use, there are no previous prospective studies designed to quantify the effect of NMP on organ utilisation. NMP is a resource-intensive alternative to SCS: high level evidence is important if this technology is to be funded by healthcare payers. There is a need to quantify the magnitude of this potential utilisation benefit in an appropriately-powered study in a real-world setting. The Perfused Liver Utilisation Study (PLUS) was designed to assess the impact of NMP availability (as opposed to NMP use) on the utilisation of livers at high risk of non-use in the UK transplant setting.

## Materials and methods

### Study design

The trial evaluated the effect of NMP availability on “functional utilisation”: the proportion of donor liver offers resulting in a functioning transplant at 12 months. To identify livers at greatest risk of non-use, a novel, regression-based score was developed using UK Transplant Registry (UKTR) data to estimate the probability of non-utilisation of individual livers offered for transplant (SDC1). This algorithm, the Donor Utilisation Index (DUI), identified the 60% of donors livers at greatest risk of decline for inclusion in the study [13].

PLUS was originally conceived as a randomised controlled trial, with eligible liver offers randomised to having NMP available or not. During sequential investigators’ meetings, surgeons in most centres became increasingly unwilling to randomise high-risk liver cohorts (particularly DCD) to SCS, prefering some form of machine preservation. The study was therefore revised to a “threshold-crossing” design, in which a real-world control cohort, selected consecutively according to the same objective inclusion/exclusion criteria to minimise bias, was used *a priori* to estimate the counterfactual (SCS cohort). A threshold was set for the primary outcome prior to recruiting a prospective single-arm study cohort (NMP available cohort) [14]. The lower bound of the 95% confidence interval for effect size had to exceed the pre-defined efficacy threshold to demonstrate superiority.

The study protocol was prospectively registered (16th November 2021; ISRCTN11552402). Ethical approval was granted by the South Central – Oxford C Research Ethics Committee (ref 21/SC/0297). Patients on the liver transplant list, liver recipients and family members were consulted during study design. A patient representative was part of the trial management group and trial steering committee, advising on conduct and dissemination.

### Inclusion/exclusion criteria

Eligible liver offers were DBD or DCD livers from UK donors aged 16 years or over, with a DUI >0.27, offered to any of the 7 UK liver transplant centres. This DUI threshold was selected as it identified the 60% most poorly utilised livers, with utilisation of 26% compared to 85% in livers with a DUI ≤0.27. Eligible offers were predominantly DCD livers (79%), reflecting lower utilisation compared to DBD livers. Offers from donors with HIV or hepatitis C were excluded, as were livers undergoing any other form of *ex-situ* machine perfusion or where the participating centre was unable to offer NMP at the time due to logistical reasons (unavailability of device, consumables or trained staff). Livers from donors undergoing NRP were eligible, with planned adjustment for NRP use in analysis. All eligible livers were enrolled to ensure comparability between cohorts.

Eligible recipients were adults (≥18 years) on the elective or super-urgent transplant waiting list. Those declining NMP use through local consent policy, receiving a split liver or reduced liver or multiorgan transplant were excluded.

### Real-world control cohort

To estimate the counterfactual, the primary outcome was assessed in a real-world control cohort where NMP was not routinely available, derived from the UKTR between 1st January 2018 and 31st December 2019 (SDC2). Identical inclusion/exclusion criteria were applied to this cohort as to the prospective study cohort, with stratified random sampling used to exclude a small proportion of patients to mimic the consent process used in the prospective cohort. A predefined efficacy threshold of at least 40 additional functional transplants per year was set, equivalent to an odds ratio of 1.22 for functional utilisation. This threshold was agreed by all seven participating centres to represent the minimum clinically-important increase in transplant rates that would justify the increased cost and logistical complexity of NMP use.

### Study cohort

A prospective cohort was recruited between 11th April 2022 and 3rd April 2023, with NMP made available for all eligible liver offers. Participating centres were informed of NMP availability at the time of offer, and the impact of availability on the primary outcome was assessed by comparing the two cohorts against the pre-defined efficacy threshold.

NMP was used at the discretion of the implanting surgeon. All participating centres had access to an OrganOx metra device and disposable kits at no additional cost, with limited per-case funding provided for staff time. NMP could be used either from the donor centre (device-to-donor) or following SCS and transport to the recipient centre (back-to-base) at the preference of the implanting team. The OrganOx metra device was used according to the current instructions for use and local protocols, with a minimum of 4 h and maximum of 24 h of perfusion time.

### Primary outcome

The primary outcome was defined as the proportion of livers with “functional utilisation”: transplantation of an offered liver with 1-year graft survival. This ensures that any increase in utilisation does not occur at the cost of inferior graft outcomes.

### Secondary outcomes

Pre-specified secondary outcomes included: proportion of non-utilisation, median biochemical liver function in the first 7 days post-transplant [bilirubin, GGT, ALT, INR, lactate (recorded during high-level care only)], and 12 months post-transplant (bilirubin, ALP, ALT, AST), median Model for Early Allograft Function score (MEAF), 12-month graft and patient survival, proportion of transplants with primary non-function, median length of ITU and hospital stay, proportion of recipients requiring renal replacement therapy after transplant and proportion of recipients experiencing adverse events in the first 3 months and 12 months after transplant. Outcomes for both cohorts were collected from the UK Transplant Registry (UKTR), with additional outcomes collected in study case report forms for the prospective cohort over a 12-month follow-up period. Unexpected serious adverse events during the 12-month follow-up period were collected for the prospective cohort only. A health-economic analysis has been undertaken and will be presented in a separate manuscript.

### Sample size and statistical design

The sample size assumed a 50% increase in 1-year functional utilisation from 21.8% in the control cohort to 32.7% in the prospective NMP available cohort, representing 135 additional successful transplants per year. Allowing for cross-over for those in the prospective cohort not receiving NMP the study was powered to detect an increase in functional utilisation to 31.2%. To achieve 90% power using a 2.5% one-sided significance level to detect an increase of at least 40 additional transplants per year, 1,035 liver offers were required. This allowed for up to 25% loss due to logistical constraints, and 10% loss due to post-recruitment exclusions and loss to follow-up. Protocol v1.0 planned a smaller sample size of 799 liver offers, but this was revised in v2.0 to allow for higher loss to follow-up and NMP unavailability for logistical reasons. An embedded feasibility study assessed recruitment rates and NMP use at 6 months, but there was no formal interim outcome analysis.

Primary analysis was by modified intention-to-treat, where livers unable to be perfused for logistic reasons were excluded, along with any found to be ineligible after study entry (once recipient characteristics were known). Analyses were adjusted for donor type and use of NRP (DBD, DCD with NRP, DCD without NRP) as a fixed effect as these both have an important effect on organ utilisation.

Primary outcome results, from logistic regression models, are reported as odds ratios (OR) with 95% confidence intervals (CI), and p-values for the cohort effect under the null hypothesis of no difference. Pre-planned subgroup analyses for the primary outcome investigated the effect of donor type, donor liver index (DLI, a UK-derived algorithm that predicts transplant outcome based on donor parameters [15]) and DUI.

Sensitivity analyses and secondary outcome analyses are described in SDC3.

All analyses were performed in SAS v9.4M5.

## Results

### Study cohorts and demographics

**FIGURE 1:**
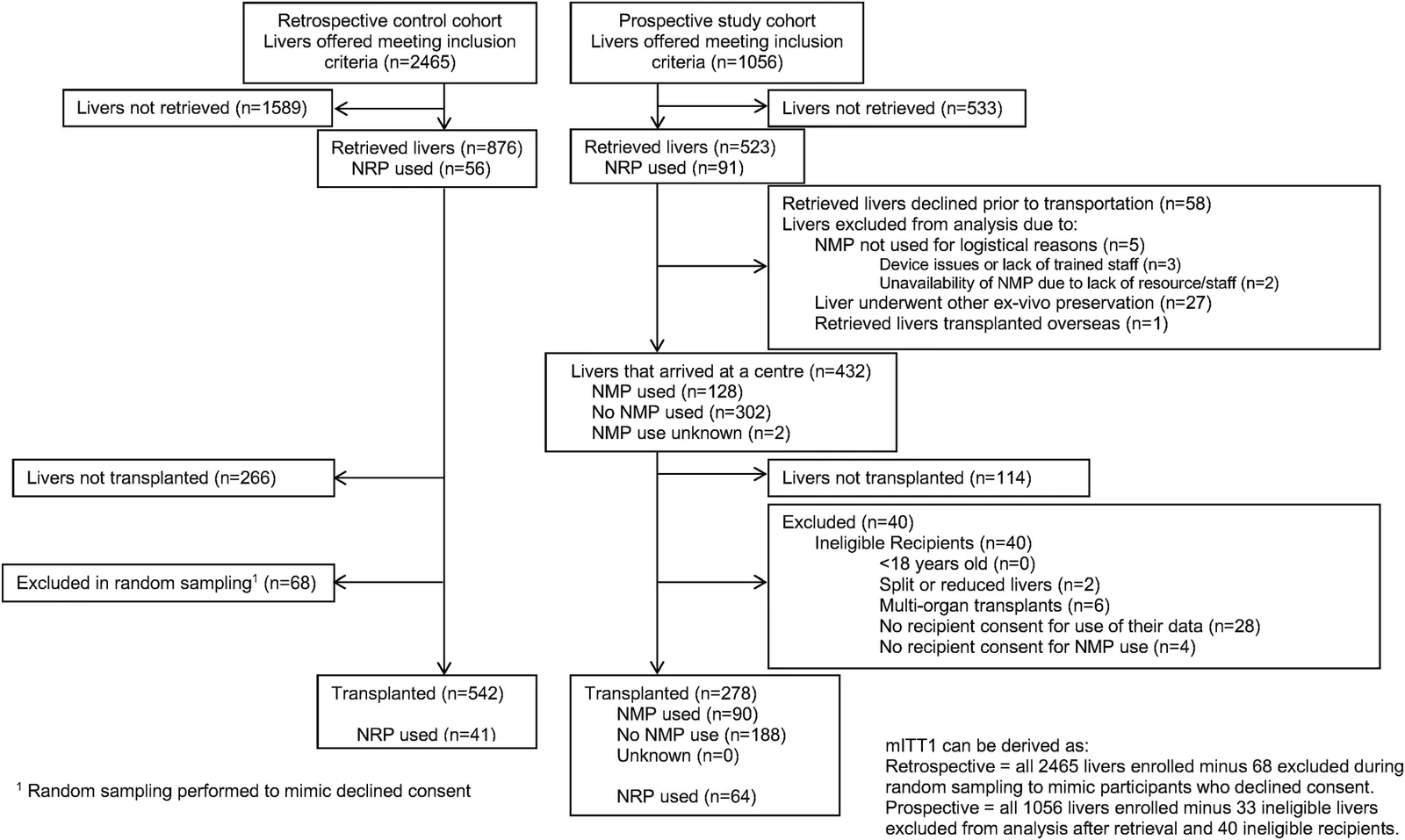
Consort flow diagram to show the flow of liver offers through the PLUS study. SCS, static cold storage; NMP, normothermic machine perfusion.

The flow of liver offers through the study is shown in Figure 1. The control cohort comprised 2,465 liver offers where SCS was the routine method of preservation and NMP was unavailable. The prospective study cohort comprised 1,056 liver offers. Of this cohort, 983 met the eligibility criteria for the modified intention-to-treat analysis. 542 control offers and 278 study offers resulted in transplants.

Donor, liver and recipient characteristics were comparable between the two cohorts (Tables 1–3) and median follow-up time post-transplant was 365 days for both cohorts. For those livers resulting in a transplant, there was an increase in the proportion of DCD livers in the prospective cohort (61.2% vs. 51.5%), indicating an increased willingness to transplant DCD livers where machine perfusion is available. Use of NRP was also higher (29.0% vs. 12.4%), reflecting changes in practice as the prospective cohort was more recent than the control cohort.

**TABLE 1:** Donor characteristics for enrolled and transplanted livers.

	Enrolled livers			Transplanted livers		
Donor characteristic	Retrospective (SCS) (n = 2,397)	Prospective (NMP available) (n = 983)	Total (n = 3,380)	Retrospective (SCS) (n = 542)	Prospective (NMP available) (n = 278)	Total (n = 820)
Age (years)	60 (51–69)	60 (51–68)	60 (51–69)	58 (48–68)	58 (46–65)	58 (48–67)
Sex						
Male Female	1,454/2,397 (60.7) 943/2,397 (39.3)	614/983 (62.5) 369/983 (37.5)	2,068/3,380 (61.2) 1,312/3,380 (38.8)	311/542 (57.4) 231/542 (42.6)	162/278 (58.3) 116/278 (41.7)	473/820 (57.7) 347/820 (42.3)
BMI (kg/m^2^)	27.8 (24.2–32.0)	28.1 (24.3–31.9)	28.0 (24.2–31.9)	27.1 (23.6–31.3)	27.7 (23.4–30.9)	27.4 (23.6–31.2)
Ethnic origin						
Asian Black White Other	56/2,375 (2.4) 25/2,375 (1.1) 2,249/2,375 (94.7) 45/2,375 (1.9)	27/959 (2.8) 13/959 (1.4) 909/959 (94.8) 10/959 (1.0)	83/3,334 (2.5) 38/3,334 (1.1) 3,158/3,334 (94.7) 55/3,334 (1.6)	19/536 (3.5) 9/536 (1.7) 495/536 (92.4) 13/536 (2.4)	6/274 (2.2) 7/274 (2.6) 259/274 (94.5) 2/274 (0.7)	25/810 (3.1) 16/810 (2.0) 754/810 (93.1) 15/810 (1.9)
Donor type						
DBD DCD	519/2,397 (21.7) 1,878/2,397 (78.3)	201/983 (20.4) 782/983 (79.6)	720/3,380 (21.3) 2,660/3,380 (78.7)	263/542 (48.5) 279/542 (51.5)	108/278 (38.8) 170/278 (61.2)	371/820 (45.2) 449/820 (54.8)
Smoker	1,552/2,383 (65.1)	593/979 (60.6)	2,145/3,362 (63.8)	334/541 (61.7)	173/276 (62.7)	507/817 (62.1)
History of diabetes	339/2,377 (14.3)	151/973 (15.5)	490/3,350 (14.6)	79/538 (14.7)	34/277 (12.3)	113/815 (13.9)
History of tumour	178/2,365 (7.5)	65/971 (6.7)	243/3,336 (7.3)	44/537 (8.2)	18/274 (6.6)	62/811 (7.6)
Cardiac/respiratory arrest	1,191/2,334 (51.0)	477/958 (49.8)	1,668/3,292 (50.7)	271/532 (50.9)	136/274 (49.6)	407/806 (50.5)
Cause of death						
CVA Anoxia Trauma Other	1,080/2,298 (47.0) 909/2,298 (39.6) 48/2,298 (2.1) 261/2,298 (11.4)	454/947 (47.9) 371/947 (39.2) 18/947 (1.9) 104/947 (11.0)	1,534/3,245 (47.3) 1,280/3,245 (39.4) 66/3,245 (2.0) 365/3,245 (11.2)	282/524 (53.8) 170/524 (32.4) 11/524 (2.1) 61/524 (11.6)	140/272 (51.5) 103/272 (37.9) 5/272 (1.8) 24/272 (8.8)	422/796 (53.0) 273/796 (34.3) 16/796 (2.0) 85/796 (10.7)
Length of ITU stay (days)	3.4 (1.8–5.8)	3.8 (2.2–5.9)	3.5 (1.9–5.8)	2.4 (1.4–4.0)	3.1 (1.9–4.6)	2.6 (1.5–4.2)
Donor utilisation index^1^						
DBD DCD	0.41 (0.33–0.55) 0.76 (0.59–0.89)	0.40 (0.31–0.54) 0.77 (0.61–0.89)	0.41 (0.32–0.55) 0.76 (0.60–0.89)	0.38 (0.32–0.48) 0.61 (0.46–0.75)	0.35 (0.29–0.44) 0.62 (0.47–0.76)	0.37 (0.31–0.47) 0.61 (0.46–0.75)
Donor liver index^2^						
DBD DCD	1.28 (1.10–1.42) 2.28 (1.99–2.57)	1.21 (1.06–1.36) 2.25 (1.99–2.53)	1.25 (1.10–1.40) 2.27 (1.99–2.56)	1.25 (1.09–1.40) 2.10 (1.81–2.45)	1.20 (1.04–1.38) 2.11 (1.91–2.37)	1.23 (1.09–1.39) 2.10 (1.84–2.41)

*Summary of missing data:*

*Enrolled livers:* Age, sex, BMI, donor type, DUI, 0; Ethnic origin, 46 (22 SCS, 24 NMP available); Smoking status, 18 (14 SCS, 4 NMP available); History of diabetes, 30 (20 SCS, 10 NMP available); History of tumour, 44 (32 SCS, 12 NMP available); Cardiac/respiratory arrest, 88 (63 SCS, 25 NMP available); Cause of death, 135 (99 SCS, 36 NMP available); Length of ITU, stay, 22 (13 SCS, 9 NMP available); DLI, 25 donors (3 DBD SCS, 18 DCD SCS, 2 DBD NMP available, 2 DCD NMP available).

*Transplanted livers:* Age, sex, BMI, donor type, DUI, 0; Ethnic origin, 10 (6 SCS, 4 NMP- available); Smoking status, 3 (1 SCS, 2 NMP available); History of diabetes, 5 (4 SCS, 1 NMP available); History of tumour, 9 (5 SCS, 4 NMP available); Cardiac/respiratory arrest, 14 (10 SCS, 4 NMP available); Cause of death, 24 (18 SCS, 6 NMP available). Length of ITU, stay, 4 (2 SCS, 2 NMP available). DLI, 4 (0 DBD SCS, 2 DCD SCS, 2 DBD NMP available, 0 DCD NMP available).

Data are n/N (%) for categorical variables, and median (IQR) for continuous variables. SCS, static cold storage; NMP, normothermic machine perfusion; BMI, body mass index; DBD, donor after brainstem death; DCD, donor after circulatory death; CVA, cerebrovascular accident.

1 See SDC for definition of the donor utilisation index (Mehew et al. [13]).

2 UK donor liver index, Collett et al. [15].

**TABLE 2:** Retrieval and perfusion characteristics for enrolled and transplanted livers.

	Enrolled			Transplanted		
Liver characteristic	Retrospective (SCS) (n = 2,397)	Prospective (NMP available) (n = 983)	Total (n = 3,380)	Retrospective (SCS) (n = 542)	Prospective (NMP available) (n = 278)	Total (n = 820)
Livers retrieved	808/2,397 (33.7)	450/983 (45.8)	1,258/3,380 (37.2)			
Degree of steatosis^1^						
Mild Moderate Severe	283/497 (56.9) 180/497 (36.2) 34/497 (6.8)	152/299 (50.8) 114/299 (38.1) 33/299 (11.0)	435/796 (54.6) 294/796 (36.9) 67/796 (8.4)	210/298 (70.5) 83/298 (27.9) 5/298 (1.7)	105/157 (66.9) 50/157 (31.8) 2/157 (1.3)	315/455 (69.2) 133/455 (29.2) 7/455 (1.5)
NRP used^2^	54/436 (12.4)	84/290 (29.0)	138/726 (19.0)	41/279 (14.7)	64/170 (37.6)	105/449 (23.4)
Cold ischaemic time (hours)				7.9 (6.4–9.5)	7.3 (6.0–9.3)	7.7 (6.3–9.5)
NMP used^3^		122/392 (31.1)	122/392 (31.1)		90/278 (32.4)	90/278 (32.4)
NMP used (% enrolled)		122/983 (12.4)	122/983 (12.4)			
NMP approach						
Back to base Device to donor		116/120 (96.7) 4/120 (3.3)	116/120 (96.7) 4/120 (3.3)		84/88 (95.5) 4/88 (4.5)	84/88 (95.5) 4/88 (4.5)
Time between donor perfusion and NMP start (hours)		6.5 (5.4–7.5)	6.5 (5.4–7.5)		6.4 (5.0–7.1)	6.4 (5.0–7.1)
Time on NMP (hours)						
Median (IQR)		7.4 (5.7–10.2)	7.4 (5.7–10.2)		8.1 (6.1–10.5)	8.1 (6.1–10.5)
Range		1.4–21.8	1.4–21.8		3.1–21.8	3.1–21.8
Categories						
≤4 h 4 - ≤8 h 8 - ≤12 h >12 h		6/119 (5.0) 57/119 (47.9) 39/119 (32.8) 17/119 (14.3)	6/119 (5.0) 57/119 (47.9) 39/119 (32.8) 17/119 (14.3)		3/87 (3.4) 40/87 (46.0) 30/87 (34.5) 14/87 (16.1)	3/87 (3.4) 40/87 (46.0) 30/87 (34.5) 14/87 (16.1)
Time between removal from NMP and recipient reperfusion (hours)^4^					0.9 (0.7–1.1)	0.9 (0.7–1.1)

*Summary of missing data (where expected):*

*Enrolled livers:* Retrieval status, NRP usage, NMP usage, 0; Steatosis, 7 (5 SCS, 2 NMP available); Degree of steatosis, 2 (1 SCS, 1 NMP available); NMP approach, Time between donor perfusion and NMP, 2; NMP duration, 3; Time between NMP and recipient reperfusion, 42.

*Transplanted livers:* NRP usage, NMP usage, 0; Steatosis, 4 (2 SCS, and 2 NMP available); Degree of steatosis, 2 livers (1 SCS, 1 NMP available); Cold ischaemia time, 9 (3 SCS, 6 NMP available); NMP approach, Time between donor perfusion and NMP, 2; NMP duration, 3; Time between NMP and recipient reperfusion, 10.

Data are n/N (%) for categorical variables, and median (IQR) for continuous variables. SCS, static cold storage; NMP, normothermic machine perfusion, NRP, normothermic regional perfusion.

1 Degree of steatosis is only reported for livers reported as steatotic.

2 Denominator is retrieved DCD livers.

3 Denominator is livers received by a centre.

4 Arterial or portal reperfusion, whichever occurred first.

**TABLE 3:** Recipient characteristics (pre-transplant).

Recipient characteristic	Retrospective control (SCS) (n = 542)	Prospective study (NMP available) (n = 278)	Total (n = 820)
Age (years)	57 (50–63)	58 (49–63)	58 (50–63)
Sex			
Male	372/542 (68.6)	196/272 (72.1)	568/814 (69.8)
Female	170/542 (31.4)	76/272 (27.9)	246/814 (30.2)
BMI (kg/m^2^)	28.4 (25.0–32.4)	28.8 (24.4–32.0)	28.4 (24.8–32.3)
Ethnic origin			
Asian	27/533 (5.1)	20/250 (8.0)	47/783 (6.0)
Black	6/533 (1.1)	7/250 (2.8)	13/783 (1.7)
White	480/533 (90.1)	219/250 (87.6)	699/783 (89.3)
Other	20/533 (3.8)	4/250 (1.6)	24/783 (3.1)
Urgency status			
Elective	523/542 (96.5)	264/272 (97.1)	787/814 (96.7)
Super-urgent	19/542 (3.5)	8/272 (2.9)	27/814 (3.3)
HCV status positive	65/530 (12.3)	17/269 (6.3)	82/799 (10.3)
Encephalopathy present	202/539 (37.5)	90/264 (34.1)	292/803 (36.4)
Renal support	25/541 (4.6)	21/269 (7.8)	46/810 (5.7)
Primary liver disease			
Acute hepatic failure	14/540 (2.6)	6/272 (2.2)	20/812 (2.5)
Autoimmune and cryptogenic disease	29/540 (5.4)	14/272 (5.1)	43/812 (5.3)
Cancer	146/540 (27.0)	44/272 (16.2)	190/812 (23.4)
Fatty liver disease – non-alcohol related	62/540 (11.5)	42/272 (15.4)	104/812 (12.8)
Fatty liver disease – alcohol related	133/540 (24.6)	75/272 (27.6)	208/812 (25.6)
Hepatitis B	8/540 (1.5)	5/272 (1.8)	13/812 (1.6)
Hepatitis C	12/540 (2.2)	6/272 (2.2)	18/812 (2.2)
Metabolic liver disease	8/540 (1.5)	5/272 (1.8)	13/812 (1.6)
Primary sclerosing cholangitis	36/540 (6.7)	35/272 (12.9)	71/812 (8.7)
Primary biliary cholangitis	39/540 (7.2)	13/272 (4.8)	52/812 (6.4)
Regraft	29/540 (5.4)	12/272 (4.4)	41/812 (5.0)
Other	24/540 (4.4)	15/272 (5.5)	39/812 (4.8)
UK end stage liver disease (UKELD score) prior to transplant^1^			
Elective	53 (50–57)	54 (50–58)	53 (50–57)
Super-urgent^3^	61 (60–64)	62 (60–67)	61 (60–64)
Model for end stage liver disease (MELD score) prior to transplant^2^			
Elective	13 (10–17)	15 (11–19)	14 (10–18)
Super-urgent^3^	36 (31–40)	24 (22–38)	35 (24–39)
INR	1.3 (1.2–1.6)	1.4 (1.2–1.7)	1.3 (1.2–1.6)
Creatinine (µmol/L)	75 (62–93)	74 (60–91)	75 (61–92)
Bilirubin (µmol/L)	36 (19–79)	41 (20–86)	37 (19–81)
Sodium (mmol/L)	137 (134–140)	137 (134–140)	137 (134–140)

*Summary of missing data:*

Age, 0; Sex, BMI, and urgency status, 6 (0 SCS, 6 NMP available); Ethnic origin, 37 (9 SCS, 28 NMP available). HCV status, 21 (12 SCS, 9 NMP available); Presence of encephalopathy, 17 (3 SCS, 14 NMP available); Renal support,10 (1 SCS, 9 NMP available); Primary liver disease 8 (2 SCS, 6 NMP available); UKELD, 34 elective (30 SCS, 4 NMP available), 2 super-urgent (1 SCS, 1 NMP available); MELD, 34 elective (30 SCS, 4 NMP available), 2 super-urgent (1 SCS, 1 NMP available); INR, 40 (29 SCS, 11 NMP available); Creatinine, bilirubin, and sodium, 9 (1 SCS, 8 NMP available).

1 UKELD, 5.395 ln (INR) + 1.485 ln (creatinine μmol/L) + 3.130 ln (bilirubin μmol/L)– 81.565 ln (sodium mmol/L) + 435.

2 MELD, 9.57 ln (creatinine mg/dL) + 3.78 ln (bilirubin mg/dL) + 11.2 ln (INR) + 6.43.

3 Small number of super urgent recipients (25 total, 18 in SCS, and 7 in NMP available).

Data are n/N (%) for categorical variables, and median (IQR) for continuous variables. SCS, static cold storage; NMP, normothermic machine perfusion; BMI, body mass index; HCV, Hepatitis C virus; UKELD, UK End stage Liver Disease; MELD, Model for End stage Liver Disease; INR, international normalised ratio.

### NMP use

In the prospective study cohort NMP was used for 31.1% of eligible livers that arrived at a transplant centre (12.4% of all livers offered). Of those where NMP was used, four livers (3.3%) used a device to donor approach, and 116 (96.7%) used a back to base approach. The approach was unknown for two livers (Table 2). There was considerable variation in NMP use between centres (SDC4) and the key reasons for non-use of NMP related to (i) livers being deemed untransplantable; (ii) surgeon deemed the liver clinically suitable for transplant without perfusion; and (iii) perfusion kit/staff unavailability (SDC10).

### Primary outcome

**FIGURE 2:**
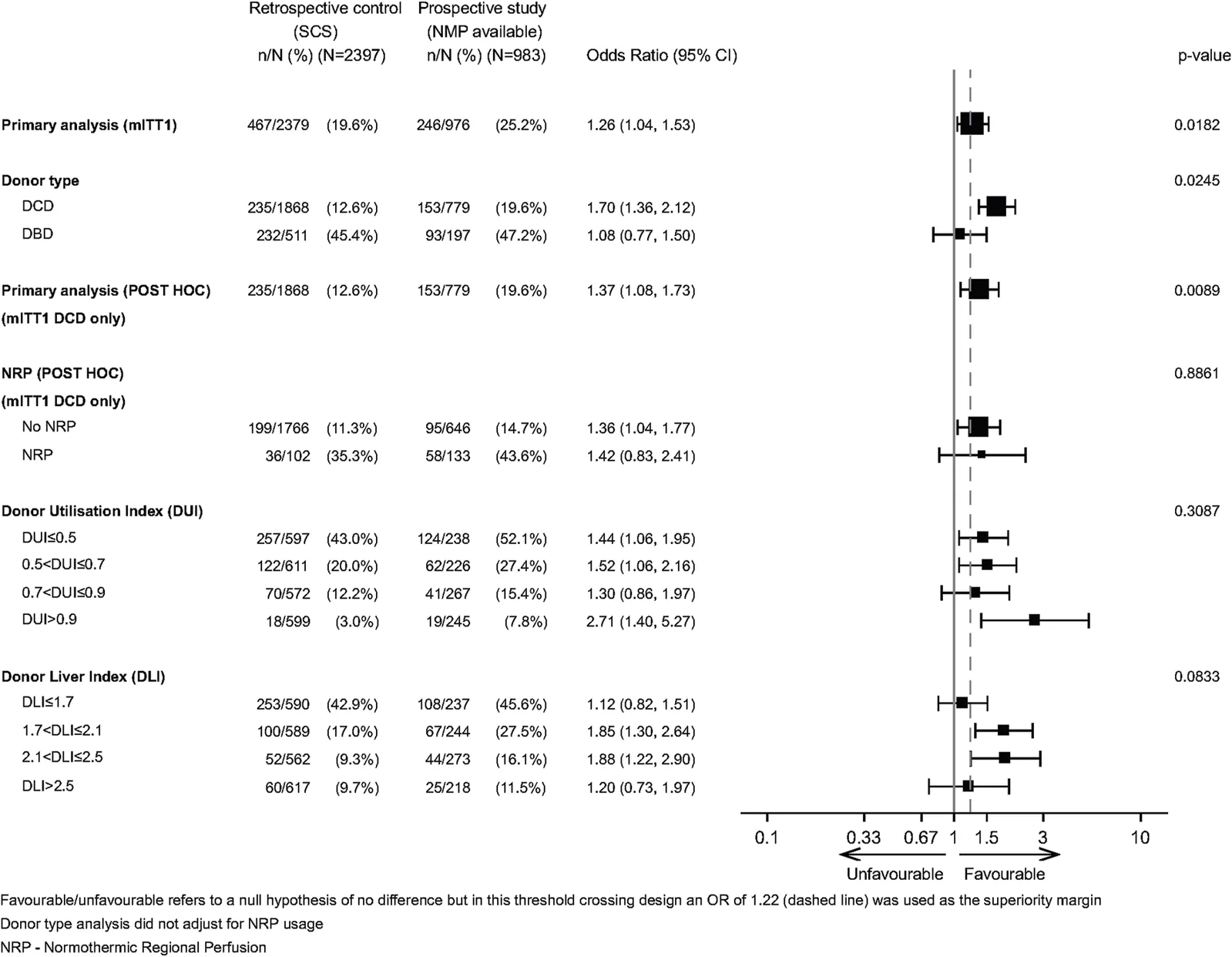
Forest plot showing the primary endpoint (functional utilisation) and planned subgroup analyses. SCS, static cold storage; NMP, normothermic machine perfusion; CI, confidence interval; DBD, donor after brainstem death; DCD, donor after circulatory death; DUI, donor utilisation index; DLI, donor liver index; NRP, normothermic regional perfusion; mITT, modified intention-to-treat.

The primary outcome of functional utilisation was significantly higher in the prospective study cohort (NMP available) (25.2% vs. 19.6%, OR: 1.26, 95% CI: 1.04–1.53), representing 47 additional functional transplants per year (95% CI 8–90 transplants) (Figure 2). However, the lower bound of the 95% confidence interval did not exceed the pre-defined threshold of 40 additional liver transplants per year, therefore the intervention is not considered superior under the threshold-crossing design. The difference in functional utilisation was primarily due to increased organ utilisation in the cohort with NMP available (27.8% vs. 22.0%) with smaller improvements in patient (95.1% vs. 92.9%) and graft (90.8% and 89.1%) survival. Reasons for non-use of livers transported to the recipient centre for the prospective cohort are shown in SDC12; the most common reason for non-use in NMP treated livers was poor function on the device (78.1%).

Subgroup analyses demonstrated a statistically significant interaction when looking at the impact of NMP availability by donor type (p = 0.0245), with a greater difference in functional utilisation observed in the DCD group (OR: 1.70, 95% CI: 1.36–2.12), exceeding the efficacy threshold. However, this analysis did not include adjustment for NRP, which is not used for DBD donors. In a *post hoc* analysis which only included DCD donors and with risk adjustment for NRP the OR was 1.37 (95% CI 1.08–1.73; p = 0.0089), not exceeding the efficacy threshold. In livers from DCD donors, *post hoc* analysis demonstrated no significant interaction between the impact of NMP and use of NRP (p = 0.8861).

**FIGURE 3:**
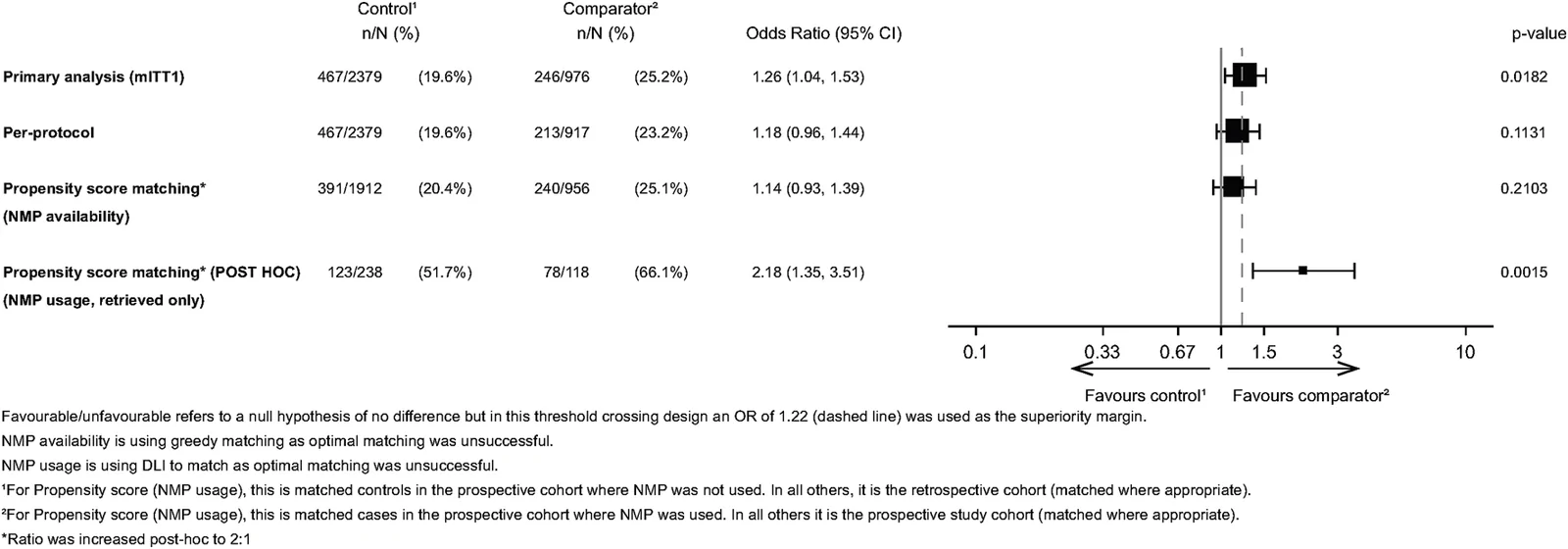
Forest plot showing sensitivity analysis for the primary endpoint (functional utilisation). SCS, static cold storage; NMP, normothermic machine perfusion; CI, confidence interval; mITT, modified intention to treat; DBD, donor after brainstem death; DCD, donor after circulatory death; DUI, donor utilisation index; DLI, donor liver index.

There were no statistically significant interactions between NMP availability and the DUI (p = 0.3087) or DLI (p = 0.0833), although for some subgroups the lower bound of the 95% confidence interval did exceed the efficacy threshold (Figure 2). A per-protocol analysis also did not exceed the efficacy threshold (OR: 1.18, 95% CI: 0.96–1.44). Two secondary, pre-specified propensity score matched analyses were also undertaken. The first mirrored the primary analysis and assessed the effect of NMP availability on functional utilisation of offered livers, and this also did not exceed the efficacy threshold (OR: 1.14, 95% CI: 0.93–1.39) (Figure 3). The second propensity score matched analysis instead assessed the impact of NMP usage (rather than availability) on functional utilisation. Post-hoc, this analysis was amended to avoid the bias whereby only retrieved livers could have received NMP and therefore analysed only retrieved livers in the prospective cohort, and compared between those where NMP was used, and propensity score matched livers where NMP was not used. This analysis did exceed the efficacy threshold, indicating that NMP does increase functional utilisation of retrieved livers when it is used (*post hoc*, OR: 2.18, 95% CI: 1.35–3.51) (Figure 3). The sample size was lower for the propensity score matched analyses than for the primary analysis.

### Secondary outcomes

There were no statistically significant differences in biochemical liver function tests at 12 months post-transplant, graft and patient survival, primary non-function, length of hospital stay, length of ITU stay, and need for renal replacement therapy (SDC5). Sensitivity analyses of graft and patient survival outcomes adjusting for recipient risk factors did also not result in statistically significant differences between the two cohorts (SDC6).

Biochemical liver function on days 1–7 and MEAF score were only available for the prospective cohort with a mean MEAF score of 4.5 (SDC8).

A larger proportion of organs were retrieved in the prospective study cohort (NMP available) compared to the retrospective control cohort (SCS) (45.8% vs. 33.7%). The proportion of organs retrieved but not transplanted was also higher in the prospective study cohort (38.2% vs. 32.9%, respectively). Following adjustment for donor type and NRP, this yielded an adjusted odds ratio for organ non-utilisation of 1.33 (95% CI: 1.04–1.71). This was statistically significant at the 5% level (p = 0.0226) (SDC9).

There was one positive perfusate culture reported. Using data collected through the UKTR the proportion of recipients experiencing infections (41.0% vs. 22.9%, respectively), treated biopsy-proven rejection episodes (19.6% vs. 16.2%, respectively), and biliary complications requiring intervention (8.7% vs. 5.1%, respectively) in the first 3 months post-transplant was higher in the retrospective control cohort (SCS) than the prospective study cohort (NMP available). Slightly lower proportions were observed in the retrospective control cohort (SCS) for vascular complications, (9.0% vs. 12.7%) and reoperations (6.4% vs. 9.0%) at 3 months post-transplant. However, data completeness varied, so no formal statistical comparisons were performed (SDC5).

### Adverse events

SAEs were only reported in the prospective study cohort (NMP available). There were 156 SAEs reported in 94 recipients (36.9% of the cohort) (SDC7). 62 SAEs were reported for cases where NMP was used, but only 1 was reported to be linked to errors in device function and use, and only 4 possibly related to the intervention.

## Discussion

The perfused liver uitlisation study (PLUS) was originally conceived as a randomised controlled trial. During study setup, clinicians became increasingly unwilling to transplant high-risk livers without perfusion, necessitating a change to a ‘threshold-crossing’ design in which a prospectively recruited cohort is compared with a retrospective real-world control cohort using the same pre-defined inclusion/exclusion criteria. All seven UK centres agreed to take part and recruitment was rapid.

PLUS demonstrated a statistically significant increase in functional utilisation of poorly utilised liver offers with availability of NMP (from 19.6% to 25.2%), but this did not exceed our pre-defined efficacy threshold. The threshold crossing design sets a higher burden of proof than a typical randomised controlled trial, requiring the lower bound of the confidence interval to exceed a predefined effect size to account for potential bias from the non-randomised design [14]. Although the effect size equates to an additional 47 successful liver transplants per year in the UK (a 4% increase), the lower confidence bound (8 transplants) did not meet the threshold of 40. Whilst the study design is sensitive to the threshold selected at the outset, these conclusions would not have changed unless the threshold has been selected to be below 8 additional transplants per year, which represents a less than 1% increase in UK transplant numbers.

The most likely explanation for the smaller than expected effect size was lower-than-anticipated uptake of NMP (12% of livers offered, 31% of eligible retrieved livers) and varied greatly between centres (ranging from 2.4% to 60.3% of eligible retrieved livers). Post-hoc propensity matched analysis suggests that when NMP is used, its effect on utilisation is greater, indicating that its full potential was likely not realised.

Several factors likely contributed to lower uptake than anticipated. Whilst the device and consumables were funded, the study budget did not cover the cost of 24/7 staffing infrastructure. In centres with existing infrastructure, NMP use was far higher than in more resource constrained centres. Similar patterns have been reported in the US, with lower use seen in low-volume centres lacking a dedicated perfusionist [16] as well as disparities related to insurance provider [17]. This disparity in access to new perfusion technologies is concerning and may have a knock-on impact on waiting times for patients in low-volume centres. Although logistical constraints were anticipated in the study design, it appears very likely that “clinician decision” was recorded as the reason not to undertake NMP in many cases where “logistical constraints” was the actual reason.

In the original European randomised controlled trial of NMP, the impact on utilisation was larger than that seen in the current study [6]. That study recruited all retrieved livers regardless of quality, with a higher baseline chance of utilisation. NMP was managed by a dedicated research fellow who travelled with the device to the donor hospital, removing the logistical burden from the receiving centre. In the PLUS study, NMP use was almost entirely back-to-base. Whilst there are no prospective randomised trials comparing the device-to-donor and back-to-base approaches, non-randomised studies suggest broadly similar outcomes [18, 19]. Uptake of NMP may be improved by provision as a service (with device and perfusionist included), or by centralisation of perfusion in dedicated Assessment and Recovery Centres (ARCs). Organs that should be considered for ARC assessment include (i) those with the highest risk of decline (perhaps defined by the DUI derived for this study) to allow further viability assessment and improve chances of utilisation, (ii) those where more time is required for logistical reasons, and (iii) organs declined by all centres, for assessment and reoffering if they meet pre-defined viability criteria. Embedding ARCs within dedicated organ recovery centres is a viable option that is already gaining traction in the US [20]. Centralised provision of perfusion technologies also has the benefit of consistent protocols and data collection, and the potential to reduce the evolving disparities in access.

Other factors affecting NMP uptake include a preference to proceed straight to transplant following SCS (with or without NRP) for lower-risk livers, or for other machine perfusion technologies including hypothermic oxygenated machine perfusion (HOPE), which is logistically much simpler. Indeed, the most common reason documented for NMP non-use during the PLUS study was clinician preference to proceed directly to transplant, although this may also partly reflect logistical/resource challenges with use of NMP. Livers undergoing HOPE were excluded from the current study, although the number was relatively small. DCD livers undergoing NRP were included, with adjustment in analysis, and NRP use increased in the prospective cohort. Notably, the effect of NMP availability exceeded our pre-defined efficacy threshold in the DCD subgroup, although this effect on utilisation was reduced when corrected for NRP use in *post hoc* analysis. This suggests that the significant improvement in functional utilisation of DCD livers seen between the two cohorts resulted from a combination of the two technologies in DCD donation. Indeed, there is already evidence from the UK that NRP can improve liver utilisation [3], and so in many cases (especially given resource constraints) additional NMP following NRP may have limited impact. However, even with the use of NRP, NMP allows additional opportunity to recondition and assess function *ex-situ* prior to transplant, and the two technologies are increasingly seen as complementary rather than exclusive particulary for livers failing NRP criteria [21, 22]. The increase in the proportion of livers retrieved, and the proportion of DCD livers transplanted, in the prospective cohort in our study suggest that with the combined use of these technologies transplant clinicians are more likely to accept organs for assessment that would previously not have been retrieved.

The liver non-utilisation rate (of retrieved livers) increased in the prospective cohort. This was accompanied by a significant increase in the liver retrieval rate, suggesting an increased willingness to retrieve and assess higher-risk organs in this cohort, possibly due to increased availability of both NMP and NRP allowing preservation and assessment.

No significant differences were seen in secondary outcomes. One-year graft and patient survival were consistent with national data, despite the cohort consisting of relatively high DLI livers. Despite an increase in the proportion of DCD livers transplanted in the NMP cohort, a smaller proportion reported biliary complications. The VITTAL study has previously suggested a high rate of non-anastomotic biliary strictures when transplanting very high-risk declined livers following use of NMP, albeit with long pre-perfusion cold ischaemic times [12]. Improved definition of cholangiocyte viability markers during NMP (including bile production, bile pH and bicarbonate levels) may help to mitigate this risk [23]. The current results suggest that in this cohort of livers at high risk of non-utilisation, functional assessment during NMP allows safe transplantation without an increase in the risk of post-transplant complications.

PLUS addressed an important question–what would the effect be of making of NMP available routinely? Strengths of the study are the real-world nature of the liver and patient cohorts, involving all UK transplant centres. The use of registry data for follow-up improved the efficiency of the study, allowing direct comparison to the retrospective control cohort.

The experience of running this study illustrates the challenges of conducting any trial of a novel (but available) intervention in which the judgement of investigators may be in flux (i.e., loss of equipoise leading to unwillingness to recruit consistently). We believe that the use of a rigorous but non-randomised design of the type used in PLUS may prove to be a useful precedent in future transplant studies. The use of a threshold-crossing design is novel in transplantation: it removes the need to randomise patients to receive conventional therapy (including no intervention), but requires the assumption that the retrospectively-recruited cohort remains typical of current practice. Indeed, one of the main weaknesses of the threshold-crossing design is that changes in practice over time may favour better outcomes in the study cohort. In PLUS, pre-study analysis demonstrated that the primary outcome of functional utilisation had remained remarkably static over time in the 4 years prior to the study, and the donor and recipient characteristics were also very similar between the cohorts. In fact, as seen in SDC12, the functional utilisation of DCD livers had been falling slightly over time, making the observation of increased utilisation in the prospective study cohort even more important. The main differences seen were in the characteristics of livers retrieved and transplanted, which are likely due to a combination of the study intervention and increased use of NRP between the cohorts, which we corrected for in the analysis.

In summary, PLUS supports the hypothesis that NMP availability increases functional utilisation of high-risk livers, but that the benefit of this new technology may be restricted unless it is properly resourced. Future implementation requires consideration of provision of NMP as a service, or centralised delivery of NMP in assessment and repair centres.

The main conclusions of this study, therefore, are that: (i) NMP availability increases organ utilisation, although less than expected; (ii) alternative trial designs are feasible and may be required where equipoise is lacking; (iii) evaluation of new technologies must include the infrastructure needed for effective implementation.

## Data Availability

The raw data supporting the conclusions of this article will be made available by the authors, without undue reservation.
