# Supplementary material for: The perfused liver utilisation study (PLUS): challenges and innovation in the evaluation of an emerging technology
Source: Transpl Int. 2026 Sep 8;39:16653. doi: 10.3389/ti.2026.16653 (PMC13593582; doi:10.3389/ti.2026.16653)
Supplement: Supplementary file 1 [file Supplementaryfile1.docx]

**Supplementary digital content**

**SDC1 – Derivation of the Donor Utilisation Index (DUI)**

The Donor Utilisation Index (DUI) is a clinical risk score that estimates the risk of non-utilisation of an offered liver, whereby only values between 0 and 1 are possible, with higher values representing higher risk of non-utilisation. It was derived from analysis of a cohort of UK liver offers from donors aged ≥16 years between 1 February 2016 and 31 January 2020. DCD donors with prolonged time to asystole were excluded. The overall sample was randomly split into model development and validation cohorts, and separate models were derived for DBD (development n=2,590, validation n=1,067) and DCD (development n=1,746, validation n=758) donors. 26 candidate variables were considered, and those statistically significant at the 10% level were included in the final model.

Both models showed good performance, with a C-statistic of 0.780 for the DBD model, and 0.796 for the DCD model. As calibration (or predictive ability) of the DBD model was comparable with that of the DCD model, DUI values from the DBD model could be compared with DUI values from the DCD model.

Note that (x)_+_ refers to the maximum of 0 and x.

*DBD Donor Utilisation Index*

$${DUI}_{DBD}=\frac{exp(\eta_{DBD})}{1+exp(\eta_{DBD})}$$

$$\eta_{DBD}=-4.1731+ \left( 0.8005 if HepB positive \right)+\left( 0.7601 if past history of diabetes \right)+\left( 0.4792 if past history of tumour \right)+0.030043 age+0.042627 bilirubin+0.005034 alk phos+\left( 0.3155 if moderate alcohol consumption \right)+\left( 1.1254 if heavy/very heavy alcohol consuption \right)-\left( 0.3342 if stopped consuming alcohol \right)+\left( 0.2501 if blood group A \right)+\left( 0.2428 if blood group B \right)+\left( 1.3751 if blood group AB \right)+\left( 0.4115 if cause of death CVA \right)-\left( 0.0484 if cause of death Anoxia \right)+\left( 0.7446 if cause of death Other \left( excluding Trauma \right) \right)+ BMIspline+ALTspline$$

$$BMIspline=-0.095502 BMI + 0.034105 F1\_BMI -0.062880 F2\_BMI$$

$$F1\_BMI=\frac{{(BMI-19.7)}_{+}^{3}-{(BMI-36.5)}_{+}^{3}}{36.5-19.7}-\frac{{(BMI-28.2)}_{+}^{3}-{(BMI-36.5)}_{+}^{3}}{36.5-28.2}$$

$$F2\_BMI=\frac{{(BMI-24.5)}_{+}^{3}-{(BMI-36.5)}_{+}^{3}}{36.5-24.5}-\frac{{(BMI-28.2)}_{+}^{3}-{(BMI-36.5)}_{+}^{3}}{36.5-28.2}$$

$$ALTspline=0.041311 ALT -0.007743 F1\_ALT +0.010706 F2\_ALT$$

$$F1\_ALT=\frac{{(ALT-11)}_{+}^{3}-{(ALT-396)}_{+}^{3}}{396-11}-\frac{{(ALT-57)}_{+}^{3}-{(ALT-396)}_{+}^{3}}{396-57}$$

$$F2\_ALT=\frac{{(ALT-25)}_{+}^{3}-{(ALT-396)}_{+}^{3}}{396-25}-\frac{{(ALT-57)}_{+}^{3}-{(ALT-396)}_{+}^{3}}{396-57}$$

*DCD Donor Utilisation Index*

$${DUI}_{DCD}=\frac{exp(\eta_{DCD})}{1+exp(\eta_{DCD})}$$

$$\eta_{DCD}=-5.2145+\left( 0.3026 if past history of smoking \right)+\left( 0.4152 if past history of diabetes \right)+\left( 0.3163 if male \right)+\left( 0.5582 if past history of tumour \right)+\left( 0.4415 if past history of hypertension \right)-(0.4145 if past history of cardiac or respiratory arrest)+ 0.000652 ALT+ 0.016575 sodium+ 0.004917 alk phos+0.002071 creatinine+\left( 0.2510 if moderate alcohol consumption \right)+\left( 1.0362 if heavy/very heavy alcohol consuption \right)-\left( 0.3321 if stopped consuming alcohol \right)+\left( 0.2293 if blood group A \right)+\left( 0.2847 if blood group B \right)+\left( 1.5143 if blood group AB \right)+\left( 0.1481 if cause of death CVA \right)+\left( 0.7689 if cause of death Anoxia \right)+\left( 0.9891 if cause of death Other \left( excluding Trauma \right) \right)+ BMIspline+agespline+ITUspline$$

$$BMIspline=0.007428 BMI + 0.029538 F1\_BMI -0.058265 F2\_BMI$$

$$F1\_BMI=\frac{{(BMI-19.9)}_{+}^{3}-{(BMI-39.7)}_{+}^{3}}{39.7-19.9}-\frac{{(BMI-29.1)}_{+}^{3}-{(BMI-39.7)}_{+}^{3}}{39.7-29.1}$$

$$F2\_BMI=\frac{{(BMI-24.8)}_{+}^{3}-{(BMI-39.7)}_{+}^{3}}{39.7-24.8}-\frac{{(BMI-29.1)}_{+}^{3}-{(BMI-39.7)}_{+}^{3}}{39.7-29.1}$$

$$agespline=0.022407 age + 0.000002 F1\_age+0.002921 F2\_age$$

$$F1\_age=\frac{{(age-26)}_{+}^{3}-{(age-76)}_{+}^{3}}{76-26}-\frac{{(age-64)}_{+}^{3}-{(age-76)}_{+}^{3}}{76-64}$$

$$F2\_age=\frac{{(age-52)}_{+}^{3}-{(age-76)}_{+}^{3}}{76-52}-\frac{{(age-64)}_{+}^{3}-{(age-76)}_{+}^{3}}{76-64}$$

$$ITUspline=-0.014439 ITU\_stay + 0.000626 F1\_ITU-0.000985 F2\_ITU$$

$$F1\_ITU=\frac{{(ITU\_stay-14)}_{+}^{3}-{(ITU\_stay-324)}_{+}^{3}}{324-14}-\frac{{(ITU\_stay-103)}_{+}^{3}-{(ITU\_stay-324)}_{+}^{3}}{324-103}$$

$$F2\_ITU=\frac{{(ITU\_stay-52)}_{+}^{3}-{(ITU\_stay-324)}_{+}^{3}}{324-52}-\frac{{(ITU\_stay-103)}_{+}^{3}-{(ITU\_stay-324)}_{+}^{3}}{324-103}$$

**SDC2 – Selection of the real-world control cohort**

The real-world control cohort was defined in advance of recruitment of the prospective study cohort in order to minimise risk of bias in selection, and to allow the pre-defined efficacy threshold to be set.

All consecutive liver offers meeting the study inclusion and exclusion criteria between 1^st^ January 2018 and 31^st^ December 2019 were identified in the UK transplant registry. This time period was contemporary enough to ensure stable utilisation over time, but excluded livers offered during the peaks of the coronavirus pandemic in the UK where offering and utilisation were affected by hospital capacity.

Broadly speaking, all livers with a donor utilisation index (DUI) greater than 0.27 from donors aged 16 years or over, offered to the seven participating UK liver transplant units, were eligible for inclusion. Those originating from or transplanted outside of the UK, or from Hepatitis C or HIV positive donors, were excluded. Livers that were split or reduced, that were transplanted as multi-organ transplants or where the recipient was aged <18 years were also excluded. Donor Utilisation Index was calculated using the formulae shown in SDC1, which differ for DBD and DCD donors. The DUI is a clinical risk score using eleven (DBD) or seventeen (DCD) donor demographic variables to predict liver graft utilisation. Recent analysis of UK donor data suggests that a cut-off of 0.27 represents the most poorly-utilised 60% of livers offered.

The Real World Control (RWC) cohort included 2465 liver offers that met all these inclusion and exclusion criteria. Records maintained by the seven participating transplant centres allowed the identification of 118 livers from this cohort (5%) in which any form of *ex-situ* machine preservation was used (including NMP and hypothermic machine preservation). These livers had lower DUI than the rest and so excluding them would bias the cohort by excluding some lower risk livers. Instead, these livers remained in the control cohort, but their outcomes were imputed to match those of other livers that were retrieved for transplantation, which had a more similar risk profile. The remaining transplanted livers in the RWC cohort would therefore have been retrieved, preserved and transported according to NHSBT national protocols. This includes placement in ice-cold perfusion solution on the back-table, and subsequently stored in cold perfusion solution within an icebox. The organ will have been transported to the recipient centre and removed from storage prior to implantation for standard back-table preparation. The duration of cold storage will have been dictated by logistics and local policy.

**SDC3 – Methodology for sensitivity and secondary outcome analyses**

Sensitivity analyses included a per-protocol analysis and propensity score matched analyses for both NMP availability (matched to retrospective livers) and use (matched to other retrieved prospective livers). The analysis population for the per-protocol analysis additionally excluded those with clinically significant protocol deviations and those who withdrew from the trial. Propensity score matching was performed using the optimal matching method with a 1:1 ratio on components of the DLI: age, donor type, smoking status, height, sex, and bilirubin. Where this was not possible, the DLI overall was used in place of its components, and the greedy method was employed if matching remained unsuccessful ^11^.

Descriptive summaries were planned for biochemical liver function in the first seven days post-transplant, MEAF, and other adverse events. For outcomes where statistical models were fitted, all analyses were adjusted for donor type and use of NRP (DBD, DCD with NRP, DCD without NRP) as a fixed effect and were further adjusted for transplant centre as a random effect (where applicable i.e. transplanted livers only). For outcomes only relevant to transplanted livers, the analysis population is further modified intention-to-treat by including only transplanted livers.

For length of stay in ITU and initial hospital stay, medians and interquartile ranges were calculated using a competing risks approach, with death as a competing risk. Cox regression analysis with a frailty term (transplant centre) was used to estimate the hazard of discharge. Graft and patient survival at 12 months post-transplant were plotted using the Kaplan-Meier approach and were also compared between cohorts using Cox regression models with frailty terms. Pre-specified sensitivity analyses additionally adjusted post-transplant survival for recipient age, MELD, BMI, and a need for renal support. Proportions of livers with primary non-function, recipients requiring renal replacement therapy, and organ non-utilisation were compared using mixed logistic regression models, except for non-utilised organs which had fixed effects only. Biochemical liver function at 12 months post-transplant was compared using mixed linear regression models, following a log-transformation to improve model fit.

No additional analyses accounting for missing data were performed; where missingness exceeded 25%, results are not presented. A sensitivity analysis for the primary outcome using multiple imputation was pre-specified if missing data for the primary outcome exceeded 5%. Data completeness is shown for each outcome.

**SDC4 – NMP use and functional utilisation by centre**

Functional utilisation (transplant with 12-month graft survival) split by whether NMP was used or not.

Please note that this is a post-hoc analysis and was not specified in the study protocol or statistical analysis plan (SAP). NMP – normothermic machine perfusion.

|  | **NMP Use** | | **Functional utilisation** | | |
| --- | --- | --- | --- | --- | --- |
| **Centre** | **Number of livers received** | **NMP usage** | **NMP not used** | **NMP used** | **Total** |
| Addenbrooke's Hospital, Cambridge | 73 | 44/73 (60.3) | 23/29 (79.3) | 27/44 (61.4) | 50/73 (68.5) |
| Freeman Hospital, Newcastle | 19 | 6/19 (31.6) | 6/13 (46.2) | 2/6 (33.3) | 8/19 (42.1) |
| King's College Hospital, London | 101 | 28/101 (27.7) | 36/71 (50.7) | 20/27 (74.1) | 56/98 (57.1) |
| Queen Elizabeth Hospital, Birmingham | 100 | 19/100 (19.0) | 58/80 (72.5) | 15/18 (83.3) | 73/98 (74.5) |
| Royal Infirmary of Edinburgh, Edinburgh | 41 | 1/41 (2.4) | 27/40 (67.5) | 1/1 (100.0) | 28/41 (68.3) |
| St James's University Hospital, Leeds | 17 | 1/17 (5.9) | 8/16 (50.0) | 1/1 (100.0) | 9/17 (52.9) |
| The Royal Free Hospital, London | 41 | 23/41 (56.1) | 9/16 (56.3) | 13/23 (56.5) | 22/39 (56.4) |
| **Total** | **392** | **122/392 (31.1)** | **167/265 (63.0)** | **79/120 (65.8)** | **246/385 (63.9)** |

**SDC5 – Secondary outcomes.** SCS – static cold storage; NMP – normothermic machine perfusion; CI – confidence interval; IQR – interquartile range; ITU – intensive care unit; IVC – inferior vena cava; AUC – area under curve. ^1^ Infections include cytomegalovirus, fungal infections and postoperative sepsis. ^2^ Biliary complications requiring intervention

| **Outcome** | **Retrospective control  (SCS)**  **(n=542)** | **Prospective study  (NMP available)**  **(n=278)** | **Total**  **(n=820)** |
| --- | --- | --- | --- |
| **Primary non-function** |  |  |  |
| N/Total N (%) | 11/542 (2.0) | 1/278 (0.4) | 12/820 (1.5) |
| Odds ratio (95% CI; P value) | **0.17 (0.02 – 1.42; p=0.1020)** | | |
| **Length of stay in ITU post-transplant (days)** | |  |  |
| N, Median (IQR) | 512, 3 (2-5) | 259, 3 (2-5) | 771, 3 (2-5) |
| Hazard ratio (95% CI; P value) | **0.97 (0.83 - 1.13; p=0.7065)** | | |
| **Length of initial hospital stay post-transplant (days)** | |  |  |
| N, Median (IQR) | 514, 13 (10-21) | 260, 14 (10-23) | 774, 14 (10-22) |
| Odds ratio (95% CI; P value) | **0.88 (0.75 - 1.03; p=0.1072)** | | |
| **Need for renal replacement therapy** | |  |  |
| N/Total N (%) | 104/511 (20.4) | 53/249 (21.3) | 157/760 (20.7) |
| Odds ratio (95% CI; P value) | **1.10 (0.75 – 1.62; p=0.6277)** | | |
| **Infections (3 months)** | |  |  |
| At least one infection^1^ - N/Total N (%) | 202/493 (41.0) | 55/240 (22.9) | 257/733 (35.1) |
| **Infections (12 months)** | |  |  |
| At least one readmission for infection - N/Total N (%) | 21/445 (4.7) | 4/213 (1.9) | 25/658 (3.8) |
| Total readmissions for infection – N | 28 | 4 | 32 |
| **Biopsy proven acute rejection (3 months)** | |  |  |
| At least one episode of rejection - N/Total N (%) | 100/511 (19.6) | 42/259 (16.2) | 142/770 (18.4) |
| Total number of episodes of rejection – N | 136 | 50 | 186 |
| **Rejection (12 months)** | |  |  |
| At least one readmission for rejection - N/Total N (%) | 14/445 (3.1) | 4/214 (1.9) | 18/659 (2.7) |
| Total number of readmissions for rejection – N | 14 | 5 | 19 |
| **Biliary complications (3 months)** | |  |  |
| At least one biliary complication^2^ - N/Total N (%) | 44/506 (8.7) | 13/257 (5.1) | 57/763 (7.5) |
| Biliary strictures – N/Total N (%) | 25/511 (4.9) | 8/258 (3.1) | 33/769 (4.3) |
| Bile duct leaks – N/Total N (%) | 23/508 (4.5) | 5/258 (1.9) | 28/766 (3.7) |
| **Biliary complications (12 months)** | |  |  |
| At least one readmission for biliary complications - N/Total N (%) | 27/445 (6.1) | 9/213 (4.2) | 36/658 (5.5) |
| Total number of readmissions for biliary complications – N | 37 | 14 | 51 |
| **Vascular complications (3 months)** | |  |  |
| At least one vascular complication - N/Total N (%) | 46/512 (9.0) | 33/259 (12.7) | 79/771 (10.2) |
| Hepatic artery thrombosis – N/Total N (%) | 9/512 (1.8) | 8/259 (3.1) | 17/771 (2.2) |
| Portal vein thrombosis – N/Total N (%) | 13/512 (2.5) | 6/259 (2.3) | 19/771 (2.5) |
| IVC or hepatic vein occlusion – N/Total N (%) | 5/512 (1.0) | 1/259 (0.4) | 6/771 (0.8) |
| Haemorrhage requiring reoperation – N/Total N (%) | 23/512 (4.5) | 19/259 (7.3) | 42/771 (5.4) |
| **Vascular complications (12 months)** | |  |  |
| At least one readmission for vascular complications - N/Total N (%) | 3/445 (0.7) | 1/213 (0.5) | 4/658 (0.6) |
| Total number of readmissions for vascular complications – N | 3 | 1 | 4 |
| **Reoperations (3 months)** |  |  |  |
| At least one reoperation in the first three months – N/Total N (%) | 32/499 (6.4) | 23/256 (9.0) | 55/755 (7.3) |
| For biliary tract stricture | 1/499 (0.2) | 2/256 (0.8) | 3/755 (0.4) |
| For bile duct leak | 10/499 (2.0) | 3/256 (1.2) | 13/755 (1.7) |
| For haemorrhage | 23/499 (4.6) | 19/256 (7.4) | 42/755 (5.6) |
| **Bilirubin (12 months) (μmol/L)** |  |  |  |
| N, Median (IQR) | 433, 11 (7**–**15) | 231, 10 (7**–**14) | 664, 10 (7**–**15) |
| Mean ratio (95% CI; P value) | **0.96 (0.86–1.07; p=0.4478)** |  |  |
| **Alkaline Phosphatase (ALP) (12 months) (IU/L)** |  |  |  |
| N, Median (IQR) | 436, 110 (85**–**164) | 231, 110 (85**–**145) | 667, 110 (85**–**157) |
| Mean ratio (95% CI; P value) | **0.94 (0.86–1.04; p=0.2196)** |  |  |
| **Alanine transaminase (ALT) (12 months) (IU/L)** |  |  |  |
| N, Median (IQR) | 374, 22 (16**–**37) | 225, 21 (14**–**31) | 599, 21 (15**–**35) |
| Mean ratio (95% CI; P value) | **0.94 (0.84–1.06; p=0.2910)** |  |  |
|  |  |  |  |

**SDC 6 – Kaplan Meier curves for graft and patient survival**

*12-month graft survival (death-censored)*

Risk adjusted hazard ratio (N included =774) 0.92 (95% CI 0.49-1.70; p=0.7783). Cox proportional hazards model adjusted for donor type, use of normothermic regional perfusion (NRP), transplant centre, recipient age, model for end-stage liver disease (MELD), body mass index and use of renal replacement therapy pre-transplant.

*12-month graft survival (non-censored)*

Risk adjusted hazard ratio (N included =774) 0.90 (95% CI 0.55-1.49; p=0.6895). Cox proportional hazards model adjusted for donor type, use of normothermic regional perfusion (NRP), transplant centre, recipient age, model for end-stage liver disease (MELD), body mass index and use of renal replacement therapy pre-transplant.

*12-month patient survival*

Risk adjusted hazard ratio (N included =774) 0.73 (95% CI 0.37-1.45; p=0.3684). Cox proportional hazards model adjusted for donor type, use of normothermic regional perfusion (NRP), transplant centre, recipient age, model for end-stage liver disease (MELD), body mass index and use of renal replacement therapy pre-transplant.

**SDC 7 – Serious adverse events.** Adverse events were only collected for the prospective cohort. NMP – normothermic machine perfusion.

|  | **Prospective study (NMP available) (n=278)** |
| --- | --- |
| Total number of events | 156 |
| Number where NMP used | 62/156 (39.7) |
| Number linked to errors in device functioning and use | 1/156 (0.6) |
| Expectedness |  |
| Not expected | 65/156 (41.7) |
| Expected | 91/156 (58.3) |
| Causality |  |
| Unrelated | 136/156 (87.2) |
| Unlikely | 16/156 (10.3) |
| Possibly | 4/156 (2.6) |
| Probably | 0/156 (0.0) |
| Definitely | 0/156 (0.0) |
| Seriousness |  |
| Hospitalisation or prolonged hospitalisation | 130/156 (83.3) |
| Likelihood of persistent or significant disability or incapacity | 0/156 (0.0) |
| Life-threatening | 12/156 (7.7) |
| Death | 7/156 (4.5) |
| Other medically significant event | 7/156 (4.5) |
| Clavien-Dindo classification |  |
| I | 21/156 (13.5) |
| II | 39/156 (25.0) |
| III | 67/156 (42.9) |
| IV | 24/156 (15.4) |
| V | 5/156 (3.2) |
| Number of participants experiencing at least one SAE (% of total number of participants) | 94/255 (36.9) |

**SDC 8 – Biochemical liver function days 1–7.** The median and IQRs for ALT on days six and seven are not reported as the values are missing for more than 25% of participants. Note that biochemistry was only collected in first 7 days post-transplant for the Prospective study cohort (NMP available). ^1^Lactate only collected when the recipient is in high level care.

|  | **Prospective study (NMP available)**  **(n=278)** | | | | | | |
| --- | --- | --- | --- | --- | --- | --- | --- |
|  | **Day 1** | **Day 2** | **Day 3** | **Day 4** | **Day 5** | **Day 6** | **Day 7** |
| **Bilirubin (μmol/L)** | | | | | | | |
| N/Total N (%) | 252/277 (91.0) | 253/276 (91.7) | 251/275 (91.3) | 252/275 (91.6) | 251/274 (91.6) | 245/274 (89.4) | 244/273 (89.4) |
| Median (IQR) | 61.0 (33.5-103.0) | 40.0 (22.0-84.0) | 43.0 (24.0-84.0) | 50.5 (26.0-89.5) | 48.0 (23.0-85.0) | 42.0 (24.0-77.0) | 34.5 (19.0-66.5) |
| **Alanine transaminase (ALT) (IU/L)** | | | | | | | |
| N/Total N (%) | 209/277 (75.5) | 211/276 (76.4) | 211/275 (76.7) | 210/275 (76.4) | 208/274 (75.9) | 205/274 (74.8) | 202/273 (74.0) |
| Median (IQR) | 621 (324-1019) | 520 (254-896) | 426 (227-708) | 338 (190-548) | 263 (155-387) | - | - |
| **INR** |  |  |  |  |  |  |  |
| N/Total N (%) | 252/277 (91.0) | 248/276 (89.9) | 251/275 (91.3) | 240/275 (87.3) | 235/274 (85.8) | 231/274 (84.3) | 225/273 (82.4) |
| Median (IQR) | 1.4 (1.3-1.8) | 1.3 (1.1-1.5) | 1.1 (1.0-1.3) | 1.1 (1.0-1.2) | 1.1 (1.0-1.2) | 1.1 (1.0-1.2) | 1.1 (1.0-1.2) |
| **Lactate (mmol/L)^1^** | | | | | | | |
| N/Total N (%) | 239/252 (94.8) | 206/222 (92.8) | 145/160 (90.6) | 106/123 (86.2) | 79/96 (82.3) | 68/79 (86.1) | 54/61 (88.5) |
| Median (IQR) | 1.3 (1.0-2.1) | 1.1 (0.8-1.5) | 0.9 (0.7-1.3) | 0.9 (0.8-1.2) | 0.9 (0.7-1.2) | 0.9 (0.8-1.3) | 1.1 (0.8-1.5) |
| **Day 1–3** | | | | | | | |
| **Model for Early Allograft Function** | | | | | | | |
| N, Mean (SD) | 211, 4.5 (2.0) | | | | | | |
|  | | | | | | | |

**SDC 9 – Rate of non-utilisation**

Note: data regarding research use is not available for the retrospective cohort.

|  | **Retrospective control (SCS) (n=2397)** | **Prospective study (NMP available)**  **(n=983)** | **Total**  **(n=3380)** |
| --- | --- | --- | --- |
| **Rate of non-utilisation** |  |  |  |
| Not transplanted – N/Total N retrieved (%) | 266/808 (32.9) | 172/450 (38.2) | 438/1258 (34.8) |
| Sent for research – n/N not transplanted (%) |  | 75/172 (43.6) |  |
| Destroyed – n/N not transplanted (%) |  | 97/172 (56.4) |  |
|  | | | |
| No NMP undertaken – n/ N not transplanted (%) |  | 140/172 (81.4) |  |
| After NMP – n/N not transplanted (%) |  | 32/172 (18.6) |  |
|  | | | |
| **Odds ratio (95% CI; P-value)** | **1.33 (1.04 - 1.71; p=0.0226)** | |  |

**SDC 10 – Reasons for not using NMP**

| **Reasons for not using NMP** | |
| --- | --- |
| **Reason** | **n/N (%)** |
| Surgeon deemed liver clinically suitable for transplant without perfusion | 177/270 (65.6) |
| Decision not to transplant prior to NMP | 74/270 (27.4) |
| Eligibility message not received | 5/270 (1.9) |
| Lack of staffing availability | 5/270 (1.9) |
| Lack of equipment | 4/270 (1.5) |
| Liver not suitable for perfusion | 1/270 (0.4) |
| Other/missing/unknown | 4/270 (1.5) |

**SDC 11 – Reasons for non-use of livers transported to a transplant centre in the prospective cohort**

| **Reasons for non-use of liver in the prospective arm treated with NMP** | |
| --- | --- |
| **Reason** | **n/N (%)** |
| **Donor unsuitable** | |
| Renal issue | 1/32 (3.1) |
| **Organ unsuitable** | |
| Poor function | 25/32 (78.1) |
| Steatosis (fatty organ) | 3/32 (9.4) |
| Poor perfusion | 2/32 (6.3) |
| **Recipient reasons** | |
| Recipient died | 1/32 (3.1) |

| **Reasons for non-use of liver in the prospective arm with SCS** | |
| --- | --- |
| **Reason** | **n/N (%)** |
| **Centre reasons** | |
| No suitable recipients | 3/79 (3.8) |
| **Donor unsuitable** | |
| Renal tumour | 2/79 (2.5) |
| Contamination | 1/79 (1.3) |
| Donor risk factors | 1/79 (1.3) |
| Renal issue | 1/79 (1.3) |
| **Logistic reasons** |  |
| NRP unavailable | 1/79 (1.3) |
| **Organ unsuitable** | |
| Steatosis (fatty organ) | 32/79 (40.5) |
| Damaged | 3/79 (3.8) |
| Fibrosis | 2/79 (2.5) |
| Ischaemia time | 2/79 (2.5) |
| Liver tumour | 2/79 (2.5) |
| Poor function | 2/79 (2.5) |
| Poor perfusion | 2/79 (2.5) |
| Contamination | 1/79 (1.3) |
| Multiple cysts | 1/79 (1.3) |
| Organ anatomy | 1/79 (1.3) |
| Organ size | 1/79 (1.3) |
| **Recipient reasons** | |
| Recipient unfit | 1/79 (1.3) |
| **Unknown** | |
| Unknown | 20/79 (25.3) |

**SDC12 – Evolution of functional utilisation prior to and during the real-world control period (2016-2019). (a) DBD liver offers; (b) DCD liver offers.**

% Functional Utilisation

| Year | DBD | DCD |
| --- | --- | --- |
| 2016 | 46% | 17% |
| 2017 | 45% | 15% |
| 2018 | 53% | 13% |
| 2019 | 46% | 12% |
